# Healthcare costs and health outcomes analysis of neoadjuvant trastuzumab therapy for human epidermal growth factor receptor 2 (HER2)-positive breast cancer

**DOI:** 10.1016/j.cpt.2025.08.006

**Published:** 2025-09-02

**Authors:** Amirhossein Jalali, Shirin Moghaddam, Andrew McGuire, Doireann P. Joyce, Bridget Carr, Diarmuid O'Leary, Emer Bourke, Ciaran O'Neill, Michael J. Kerin, Paddy Gillespie, James A.L. Brown

**Affiliations:** a School of Medicine, University of Limerick, Limerick, V94 T9PX, Ireland; b Limerick Digital Cancer Research Centre (LDCRC), University of Limerick, Limerick, V94 T9PX, Ireland; c Health Research Institute (HRI), University of Limerick, Limerick, V94 T9PX, Ireland; d Department of Mathematics and Statistics (MACSI), University of Limerick, Limerick, V94 T9PX, Ireland; e Discipline of Surgery, School of Medicine, University of Galway, H91 V4AY, Ireland; f Patient and Public Involvement (PPI) Breast Cancer Panel, University Hospital Limerick, Limerick, V94 F858, Ireland; g Discipline of Pathology, Lambe Institute for Translational Research, University of Galway, H91 V4AY, Ireland; h Centre for Chromosome Biology, Biomedical Sciences Building, School of Science, University of Galway, H91 W2TY, Ireland; i School of Medicine, Dentistry and Biomedical Sciences, Queens University Belfast, BT9 7BL, United Kingdom; j School of Business and Economics, University of Galway, H91 TK33, Ireland; k Department of Biological Science, University of Limerick, Limerick, V94 T9PX, Ireland; l Bernal Institute, University of Limerick, Limerick, V94 T9PX, Ireland

**Keywords:** Breast neoplasms, Trastuzumab, Neoadjuvant therapy, Cost-benefit analysis, Quality-adjusted life years, Cost-effectiveness analysis, Delivery of health care, Health care costs

## Abstract

**Background:**

Globally, the incidence of breast cancer continues to rise; however, mortality rates are declining due to the growing effectiveness of targeted therapies and treatments. Overexpression of human epidermal growth factor receptor 2 (*HER2*) is seen in ∼15% of breast cancers (termed *HER2^+^*). trastuzumab is the standard *HER2*-targeted therapy for *HER2^+^* breast cancers in the adjuvant setting, and is increasingly being used as a neoadjuvant chemotherapy treatment (NACT or NAC). However, as well as the clinical impact, using drugs in a different treatment setting (including neoadjuvant therapy) has a financial impact. Economic evaluation of novel chemotherapeutic strategies can assess both clinical utility and cost-effectiveness, thereby informing and guiding healthcare resource allocation decisions. Currently, the cost, clinical outcomes, and cost-effectiveness of single-agent neoadjuvant trastuzumab remain underexplored. In this study, we evaluated the cost-effectiveness of trastuzumab administered as neoadjuvant therapy, adjuvant therapy, or a combination of both regimens (NACT/ACT).

**Methods:**

A 3-year retrospective observational comparative analysis was conducted to examine costs and health outcomes using clinicopathological data (treatment type, surgical procedure, breast cancer subtype) from a public hospital in Ireland. Overall, 192 non-metastatic, non-palliative *HER2^+^* breast cancer patients (Luminal B *HER2*, and *HER2^+^* [non-luminal]) were selected (151 adjuvant trastuzumab treated, 28 neoadjuvant trastuzumab treated, 13 NACT/ACT trastuzumab treated). The analysis estimated the cost of treatment (chemotherapy regimen, surgery type) and health outcomes, which were evaluated by analysis of survival data, and by calculating quality-adjusted life years (QALYs) and average cost-effectiveness ratios (ACERs). Multivariate regression analysis, using survival regression model techniques, was performed to evaluate associations between treatment types and total costs-adjusted by age, stage, grade, and subtype. A Cox proportional hazard model estimated the effect of treatment alternatives for time to disease-free survival (DFS).

**Results:**

Multivariate analysis demonstrated no significant difference in treatment cost (*P* = 0.318), surgery cost (*P* = 0.951), or DFS (*P* = 0.236) between the adjuvant and neoadjuvant trastuzumab treatment groups. A significantly higher treatment cost was observed in older patients (*P* = 0.011) and patients with Grade 3 tumours (*P* = 0.037). No significant difference in cost was found between the *HER2* subtype groups (*P* = 0.129) or between disease stages (*P* = 0.710). No statistically significant difference in QALY was observed between adjuvant and neoadjuvant treatment groups (*P* = 0.296).

**Conclusion:**

Overall, while adjuvant trastuzumab remains the most cost-effective strategy for patients with *HER2^+^* breast cancer, adopting a neoadjuvant trastuzumab approach does not appear to pose a significant economic disadvantage. Notably, higher treatment costs were observed among older patients, a finding with important financial implications for healthcare systems. These results highlight the need for careful evaluation to inform forthcoming age-related cancer policy updates.

## Highlights

• Treatment costs were significantly increased in older patients (*P* = 0.011).
• No significant difference in quality-adjusted life years (QALYs) was observed between patients treated with adjuvant vs neoadjuvant trastuzumab.
• The average cost-effectiveness ratio (ACER) for adjuvant trastuzumab was €18,828/QALY, compared with €21,770/QALY for neoadjuvant trastuzumab.
• No significant difference in DFS (3 years) was found between the neoadjuvant and adjuvant trastuzumab groups.
• Treatment costs did not differ significantly by type of surgery, breast cancer subtype, or the interaction between subtype and treatment.

## Graphical abstract

**Figure:**
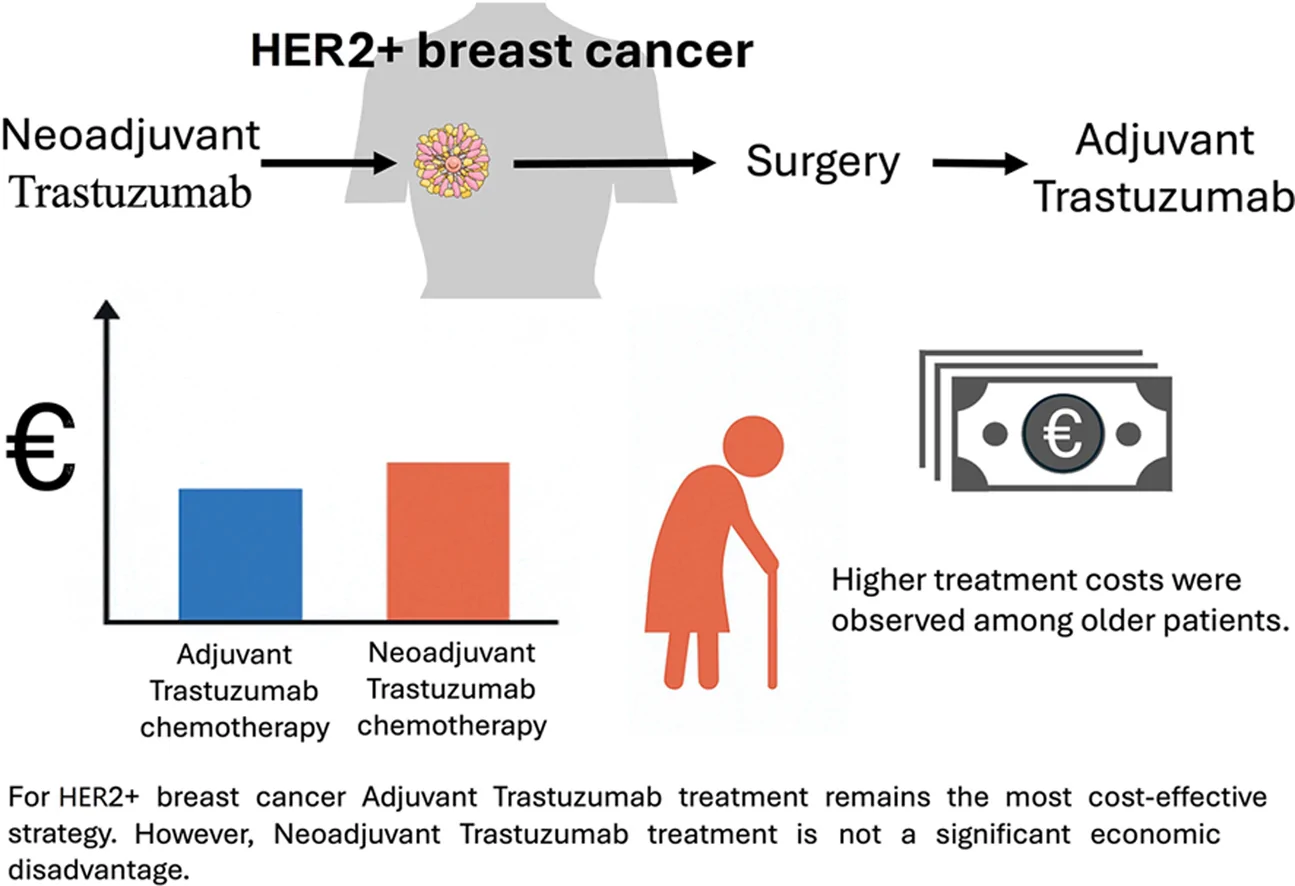

## Introduction

Despite an increasing incidence of breast cancer worldwide, the mortality rate has decreased, with age group-specific improvements in survival.^1^, ^2^, ^3^, ^4^ This reduction is driven by significant improvements in prognostic and diagnostic tests (including enhanced tumour stratification/subtyping using more precise biomarkers), and the development of highly effective and targeted/personalized treatments.^4^^,^^5^ The evaluation of new treatment strategies (once clinical effectiveness is established) includes a cost-effectiveness analysis before widespread adoption of a treatment regimen.^6^ Cost-effectiveness evaluates if a new intervention has improved patient outcomes and is financially viable in the healthcare system.^7^^,^^8^ Cost-effectiveness methodological guidelines are issued by individual regional regulatory authorities, in Ireland, the Health Information and Quality Authority (HIQA),^9^ and evaluate the relative costs and health outcomes of new treatments relative to existing therapies.

World Health Organization (WHO) guidelines recommend cost-effectiveness comparisons to existing interventions, a fixed price cut-off point or threshold value (representing the assumed social willingness to pay for an additional unit of health).^10^ This requires a health outcome measure for comparison, usually the quality adjusted life years (QALY) calculation, a utility score of disease burden including quality and quantity of life, with one QALY equating to one year of perfect health.^7^ To assess cost-effectiveness, the incremental cost-effectiveness ratio (ICER) per QALY is calculated as a ratio of incremental costs to incremental effects.^8^ Where the ICER falls below the price cut-off point/threshold value, the new treatment will be deemed cost-effective. In Ireland's Health Service Executive, cost-effectiveness thresholds are typically set between €20,000 to €45,000 per QALY, as defined by HIQA.^9^

In breast cancer, emerging clinical advances which influence the changing cost landscape include prognostic testing (e.g. Oncotype DX testing),^11^^,^^12^ targeted therapies (e.g. trastuzumab),^5^^,^^13^ optimisation of treatment timing [adjuvant or, neoadjuvant chemotherapy (NACT)],^14^^,^^15^ and surgical innovations such as sentinel lymph node biopsy (SLNB), breast-conserving surgery (BCS), and mastectomy with or without reconstruction.^16^^,^^17^ Combined with enhanced recovery programs, these advances have resulted in large reductions in length of stay (LOS) as an inpatient in hospital with a significant increase in day-case surgeries.^18^^,^^19^ These advances have markedly improved patient survival, QALY, and reduced healthcare costs.^20^, ^21^, ^22^

Human epidermal growth factor receptor 2 (*HER2*)-positive (*HER2*^+^) breast cancers can be subdivided into two distinct and clinically relevant breast cancer subtypes, Luminal B (positive for receptors: oestrogen, progesterone, *HER2*) and *HER2^+^* (non-luminal) subtypes. Importantly, both subtypes can be treated using *HER2* receptor-targeting chemotherapeutics (e.g. trastuzumab). Adjuvant trastuzumab administered post-surgery has resulted in a significant improvement in patients’ disease-free survival (DFS) and overall survival (OS),^23^, ^24^, ^25^, ^26^ and is considered cost-effective over a lifetime horizon.^20^^,^^27^^,^^28^ Recently, regimens incorporating trastuzumab in the neoadjuvant (pre-surgery) setting have been shown to improve OS and DFS.^29^^,^^30^ Newer *HER2* targeted treatment combination approaches, such as neoadjuvant Pertuzumab and adjuvant trastuzumab, have demonstrated cost-effectiveness for *HER2^+^* early breast cancer (EBC) and are associated with increased pathological complete response (pCR) rates.^31^ pCR impacts subsequent clinical management options, including the need for surgery or additional chemotherapy cycles. Conversely, patients who do not respond to neoadjuvant treatment generally require a switch to adjuvant treatment.

For EBC *HER2^+^* patients, the current National Cancer Control Program guidelines recommend trastuzumab in combination with chemotherapy, followed by 52 weeks of maintenance trastuzumab,^32^ which is associated with additional costs of approximately €25,000. Additionally, long-term trastuzumab therapy used for the treatment of metastatic and recurrent breast cancer has significant financial implications.^33^^,^^34^ Importantly, a subset of *HER2^+^* patients derive no clinical benefit from adjuvant trastuzumab, with cancer recurring in up to 7.5% of patients in the first year.^35^ Conversely, some patients experience long-term survival without trastuzumab treatment, with up to 67.1% of patients disease-free after 4 years.^23^ Cost-effectiveness analyses of adjuvant trastuzumab indicate that despite higher initial costs, it remains cost-effective.^20^^,^^24^^,^^27^ However, the cost, clinical outcomes, and cost-effectiveness of single-agent neoadjuvant trastuzumab have not been adequately studied.

We hypothesize that switching to neoadjuvant trastuzumab will not have a significant economic impact, a finding that could guide future policy decisions in an ageing cancer-presenting population increasingly receiving neoadjuvant therapy. The current study aimed to evaluate the cost-effectiveness of neoadjuvant vs adjuvant trastuzumab for treating *HER2^+^* patients in an Irish setting over 3 years of follow-up. Specifically, we assessed how trastuzumab treatment type (adjuvant or neoadjuvant), surgery type, clinicopathological features -including age of patients, tumour grade, subtype-impacted on the cost-effectiveness of using trastuzumab to treat *HER2^+^* breast cancer patients.

## Methods

### Cohort selection

The retrospective study cohort consists of all patients with non-metastatic *HER2* receptor-positive (*HER2^+^*) breast cancers treated at a tertiary referral unit (University Hospital Galway), drawn from a prospectively maintained database (2004–2016) and, with data access and analysis conducted in 2017–2018. Only patients with a definitive *HER2^+^* subtype and 3-year follow-up were included in the analysis (*n* = 192). All clinical pathological details and treatment regimens were analysed from the anonymised dataset provided. Hormone receptor-positive patients received hormone therapy, as per treatment protocols at the time of diagnosis. Patients were excluded if they had metastatic disease at diagnosis, received trastuzumab for palliative purposes only, lacked complete treatment regimen data, or were treated with additional anti-*HER2* targeting agents (e.g. Lapatinib or Pertuzumab). For analysis, patients were classified into three treatment groups: adjuvant trastuzumab, neoadjuvant trastuzumab, and a combined regimen of adjuvant and neoadjuvant trastuzumab (NACT/ACT). For each patient, a cost profile was generated, allowing cost and QALY analyses to be performed across defined patient cohorts.

### Cost analysis

A healthcare provider perspective was adopted for costing, with resource use identified and measured over the course of the 3-year follow-up period, and valued using a vector of Irish, or where necessary, UK unit cost data. All costs are reported in 2010–2012 prices, as previously described.^25^ Data is available on request. Treatment costs for all non-metastatic *HER2*-positive patients were calculated from the perspective of a public hospital healthcare provider, incorporating three-year disease-free survival (DFS). The costs of the following resource items were included: surgery, chemotherapy, radiotherapy, and hormone therapy treatments. Notably, additional costs like length of stay or ICU admission were excluded from analysis to avoid any potential double-counting or overestimation of costs.

### Quality adjusted life year analysis

The QALY over the 3-year follow-up period was estimated by assigning utility scores over the observed survival period. Utility scores for treatment and recurrence were obtained from published studies.^20^ In particular, a utility of 0.584 was assigned to metastatic breast cancer in its first year, and 0.604 thereafter; a utility of 0.725 was assigned to breast cancer recurrence in its first year, and 0.708 thereafter; and a utility of 0.728 was assigned to disease-free breast cancer in its first year, and 0.805 thereafter.^21^ The QALY was calculated for all patients with 3 years of follow-up data, excluding those patients without 3 years of follow-up data.

### Cost-effectiveness analysis

An exploratory cost-effectiveness analysis was undertaken to examine the potential cost-effectiveness of the alternative strategies under consideration (standard adjuvant trastuzumab compared to: neoadjuvant trastuzumab, or combined adjuvant & neoadjuvant trastuzumab). A series of average and incremental cost-effectiveness ratios were estimated. ACERs were estimated for the strategy by dividing its estimated mean cost by its estimated mean QALY. ICERs were estimated as the ratio of the difference in mean costs and mean QALYs between the alternative strategies. ICERs were calculated to compare neoadjuvant trastuzumab or NACT/ACT trastuzumab with the reference strategy adjuvant trastuzumab. Additionally, ICERs were calculated based on breast cancer subtype. Note that if a treatment strategy was associated with increased costs and decreased QALYs relative to its comparator, it was denoted as dominated by this comparator. Costs were reported in Euros and reflect unit costs at the time of treatment from the perspective of the Irish public healthcare system. Inflation and exchange rate adjustments were not applied, in line with standard practice for retrospective analyses.

### Statistical analysis

A series of descriptive and multivariate regression analyses, using survival and linear regression model techniques, were undertaken. Means, medians, standard deviations, and interquartile ranges were estimated to summarise costs, survival, and QALYs. Total costs were analysed using log-transformed linear regression to address skewness, adjusting for age, stage, grade, and subtype. The impact of treatment alternatives on disease-free survival time was estimated using a Cox proportional hazard model. Statistical significance was explored at the 0.05 level, model fit by the Akaike information criterion (AIC). For the exploratory cost-effectiveness analysis, a simple descriptive approach was adopted for the presentation of estimates for mean costs, mean QALYs, average cost-effectiveness ratios (ACERs), and ICERs.^8^

For the exploratory cost-effectiveness analysis, mean costs and mean QALYs were estimated by treatment group and ACERs were calculated as the ratio of mean cost to mean QALY within each group. ICERs were calculated by comparing each treatment group to the reference group of adjuvant trastuzumab using differences in mean costs and QALYs. Confidence intervals for mean QALYs were estimated using a normal approximation, while ICER confidence intervals were derived via non-parametric bootstrapping (1000 replications) to reflect uncertainty in cost and QALY estimates. A sensitivity analysis was conducted by varying utility parameters within their 95% confidence intervals reported in the published standard errors^36^ and comparing ICERs against the Irish cost-effectiveness threshold of €45,000 per QALY.^37^ For the total cost and time to disease-free survival analyses, 14 cases with missing key variables were excluded from the analysis. All analyses were performed using R (version 4.2.2).

## Results

### Cohort analysis

The study cohort comprised 192 non-metastatic breast cancer patients with a median age of 61 years [Table 1]. Of these, 79% (*n* = 151) received adjuvant trastuzumab, 15% (*n* = 28) received neoadjuvant trastuzumab and 7% of the patients (*n* = 13) received NACT/ACT trastuzumab. Radiotherapy was administered to 85% (*n* = 163) of patients, while 65% (*n* = 124) of hormone receptor-positive breast cancers received hormone therapy. pCR was recorded for 5% (*n* = 10) of patients, nine in chemotherapy-treated patients, and one in NACT/ACT-treated patients [Table 1]. Analysing 3-year DFS by treatment group, 91% (*n* = 137) of patients receiving adjuvant trastuzumab remained disease-free after 3 years, compared to 75% (*n* = 21) in the neoadjuvant trastuzumab group with all the patients in the NACT/ACT group remaining disease-free after 3 years (*n* = 13).

**Table 1:** Cohort characteristics of the study (*n* = 192).

Variable	Value
Median age (years)	61 (52–69)
Stage:	
1	*n* = 39 (20)
2	*n* = 80 (42)
3	*n* = 61 (32)
pCR	*n* = 10 (5)
Unknown	*n* = 2 (1)
*HER2^+^* breast cancer subtype	
Luminal B *HER2*	*n* = 115 (60)
*HER2^+^* (non-luminal)	*n* = 77 (40)
trastuzumab treatment group	
Adjuvant trastuzumab-treated	*n* = 151 (79)
Neoadjuvant trastuzumab-treated	*n* = 28 (15)
Neoadjuvant trastuzumab pCR	*n* = 9 (5)
NACT/ACT trastuzumab-treated	*n* = 13 (7)
pCR	*n* = 1 (5)
Radiotherapy treated	*n* = 163 (85)
Hormone therapy treated	*n* = 124 (65)

Data presented as *n* (%), or median (interquartile range, IQR). ACT: Adjuvant chemotherapy; *HER2*: Human epidermal growth factor receptor 2; NACT: Neoadjuvant chemotherapy treatment; pCR: Pathological complete response.

### Cost analysis of adjuvant or neoadjuvant trastuzumab treatment

The overall mean cost of treatment (including surgery, chemotherapy, radiotherapy and hormone therapy treatments) for the cohort (192) was €44,731 (95% CI 40,975 – 48,518) [Table 2], with the mean increasing to €59,914 in patients experiencing metastasis or breast cancer recurrence (*n* = 31). Interestingly, the mean treatment cost for patients achieving pathological complete response pCR (*n* = 10) was €44,238. The mean cost in the adjuvant trastuzumab treated group was €43,682 (95% CI, 39,260–48,103), compared with €50,093 (95% CI, 41,387–58,799) in the neoadjuvant group, and €45,367 (95% CI, 30,675–60,058) in the NACT/ACT treated group [Table 2]. Importantly, no statistically significant difference in cost *(P* = 0.318) was observed between groups.

**Table 2:** Total calculated cost by treatment group.

Treatment group	Patients (*n*)	Mean cost (€)	Value (€), 95% CI
Total cohort	192	44,731	40,945–48,516
Adjuvant trastuzumab treated	151	43,682	39,260–48,103
Neoadjuvant trastuzumab treated	28	50,093	41,387–58,799
NACT/ACT trastuzumab treated	13	45,367	30,675–60,058

All costs are reported in Euros (₠), valued at 2010–2012 prices. 95% CI: 95% confidence interval; ACT: Adjuvant chemotherapy; NACT: Neoadjuvant chemotherapy.

### Cost analysis by breast cancer surgery type

The total treatment costs were evaluated by surgery type-wide local excision (WLE) vs mastectomy [Table 3]. No significant difference (*P* = 0.951) in cost was found when comparing patients undergoing WLE (*n* = 107) €44,927 (95% CI, 41,444–48,409) and those undergoing mastectomy (*n* = 82) €44,666 (95% CI, 36,946–52,386).

**Table 3:** Total treatment cost by surgery type, tumour subtype, and treatment regimen.

Parameters	Patients (*n*)	Mean cost (€)	95% CI	*P* value
Surgery type				
WLE	107	44,927	41,444–48,409	0.951
Mastectomy	82	44,666	36,946–52,386	
Tumour subtype				
Luminal B *HER2*	115	43,822	40,632–47,011	0.612
*HER2^+^* (non-luminal)	77	45,639	35,670–55,608	
Adjuvant group				
Luminal B	91	42,391	38,910–45,872	0.540
*HER2^+^* (non-luminal)	60	44,757	40,725–48,788	
Neoadjuvant group				
Luminal B	16	50,092	42,803–57,381	0.999
*HER2^+^* (non-luminal)	12	50,094	30182–70,007	

All costs are reported in Euros (€), valued at 2010–2012 prices. 95% CI: 95% confidence interval; *HER2*: Human epidermal growth factor receptor 2; WLE: Wide local excision.

### Cost analysis by human epidermal growth factor receptor 2 (*HER2*) positive breast cancer subtype

Evaluating treatment cost by *HER2* subtype, no significant difference in cost (*P* = 0.612) was found between the Luminal B €43,822 (95% CI, 40,632–47,011) and the *HER2^+^* (non-luminal) subtypes €45,639 (95% CI, 35,670–55,608) [Table 3].

### Cost analysis by treatment group and human epidermal growth factor receptor 2 (*HER2*) subtype

When comparing costs by trastuzumab regimen (adjuvant or neoadjuvant) and subtype, no significant difference was observed (*P* = 0.540) between adjuvant trastuzumab-treated Luminal B €42,391 (95% CI, 38,910–45,872) and *HER2^+^* (non-luminal) €44,757 (95% CI, 40,725–48,788) subtypes. Similarly in neoadjuvant-treated groups, there was no significant difference (*P* = 0.999) between Luminal B costs €50,092 (95% CI, 42,803–57,381) and *HER2^+^* (non-luminal) €50,094 (95% CI, 41,818–49,193) costs [Table 3].

Among the 14 patients excluded from the total cost and disease-free survival analyses, the average age was lower than in the included cohort (53 vs 61 years). The distribution of disease stage in the excluded group was Stage I: 14.5%; Stage II: 71%; and Stage III: 14.5% compared to 22%/45%/33% respectively in the included group. Luminal B subtype was also less frequent amongst the excluded patients (43% vs 61%). Overall, these differences appear minor, and no evidence of major systematic bias was identified.

### Multivariate analysis of total costs

A non-linear regression model was developed to assess the impact of treatment regimen (adjuvant, neoadjuvant, or neoadjuvant/adjuvant) on the total cost of breast cancer treatment [Table 4]. Age demonstrated a significant effect on the total cost (*P* = 0.011), as did tumour grade (Grade 3 vs Grades 1–2; *P* = 0.037). Compared with the adjuvant-treated group, neoadjuvant trastuzumab did not have a significantly higher cost (*P* = 0.420). Similarly, cancer stage (Stage III vs Stage 1) had no significant effect on cost (*P* = 0.71).

**Table 4:** Multivariate non-linear regression for total cost.

Variable	Coef.	95%CI	*P* value
Age	0.989	0.981–0.997	0.011
Stage 2 vs. 1	0.988	0.782–1.248	0.919
Stage 3 vs. 1	1.261	0.980–1.624	0.071
Grade 3 vs. 1 & 2	1.235	1.013–1.507	0.037
Treatment group Neoadjuvant vs adjuvant	1.131	0.837–1.529	0.420
Treatment group NACT/ACT vs adjuvant	0.925	0.630–1.359	0.690
Subtype Luminal B vs *HER2^+^* (non-luminal)	1.161	0.957–1.408	0.129

95% CI: 95% confidence interval; ACT: Adjuvant chemotherapy; Coef.: Coefficient; ACT: Adjuvant chemotherapy; Coef.: Coefficient; *HER2*: Human epidermal growth factor receptor 2; NACT: Neoadjuvant chemotherapy.

### Multivariate analysis of disease-free survival

A Cox proportional hazard model for disease-free survival (DFS) was conducted [Table 5]. No significant difference in DFS was observed (*P* = 0.236) between adjuvant and neoadjuvant trastuzumab groups (HR of 1.93, 95% CI, 0.639–6.159). Similarly, no significant difference (*P* = 0.531) was seen in DFS between patients who received adjuvant/neoadjuvant trastuzumab, compared with adjuvant trastuzumab alone (HR of 1.501, 95% CI, 0.421–5.354).

**Table 5:** Cox proportional hazard model for time to disease-free survival.

Variable	Coef.	95%CI	*P* value
Age	0.984	0.951–1.018	0.356
Grade 3 vs. 2 & 1	1.081	0.472–2.478	0.853
Treatment group Neoadjuvant vs. adjuvant	1.983	0.639–6.159	0.236
Treatment group NACT/ACT vs. adjuvant	1.501	0.421–5.354	0.531
Subtype Luminal B vs. *HER2^+^* (non-luminal)	0.758	0.336–1.712	0.505

95% CI: 95% confidence interval; ACT: Adjuvant chemotherapy; Coef.: Coefficient; *HER2*: Human epidermal growth factor receptor 2; NACT: Neoadjuvant chemotherapy.

### Exploratory cost-effectiveness analysis

An exploratory analysis of the potential cost-effectiveness of adjuvant and neoadjuvant trastuzumab treatments was conducted [Table 6]. The three-year QALY value was 2.194 (95% CI, 2.131–2.258) for the adjuvant trastuzumab group, 2.112 (95% CI, 1.901–2.324) for the neoadjuvant trastuzumab group, and 2.170 (95% CI, 2.012–2.329) in the NACT/ACT group. The ACER was estimated as €18,828/QALY (17,837–22,368) for the adjuvant trastuzumab-treated group, €21,770/QALY (20,061–28,593) for the NACT/ACT-treated group, and €19,404/QALY (15,351–27,440) for the NACT/ACT group. No significant difference in QALY was seen between treatment groups (*P* = 0.662 and *P* = 0.243) [Table 6].

**Table 6:** QALY, Cost/QALY and ACER by treatment.

Treatment group	Patients (*n*)	QALYs (95%CI)	*P* value^a^	Average Cost (€)	ACERs
Adjuvant trastuzumab-treated	151	2.194 (2.131–2.258)	–	43,682	18,828 (17,837–22,368)
Neoadjuvant trastuzumab-treated	28	2.112 (1.901–2.324)	0.662	50,093	21,770 (20,061–28,593)
NACT/ACT trastuzumab-treated	13	2.170 (2.012–2.329)	0.243	45,367	19.404 (15,351–27,440)

a *P* value of the Wilcoxon rank test for determining whether QALYs differ significantly from the ‘Adjuvant trastuzumab treated’ group. All costs are reported in Euros (€), valued at 2010–2012 prices. 95% CI: 95% confidence interval; ACERs: Average cost-effectiveness ratios; HER2: Human epidermal growth factor receptor 2; NACT: Neoadjuvant chemotherapy; QALYs: Quality-adjusted life years.

The exploratory analysis of the cost-effectiveness of the alternative treatment strategies was performed [Table 7]. Relative to a ‘do nothing’ scenario, the ACER for adjuvant trastuzumab corresponds to an ICER of €18,828, establishing this as the most cost-effective strategy across all *HER2^+^* subgroups. In contrast, both neoadjuvant and NACT/ACT trastuzumab were associated with higher costs and slightly lower QALYs, resulting in negative ICERs and thus considered dominated strategies. When compared to adjuvant trastuzumab, the estimated ICERs were -€78,183/QALY for neoadjuvant trastuzumab and -€70,208/QALY for NACT/ACT trastuzumab, indicating that both strategies were, on average, more costly and less effective. Subgroup analyses yielded consistent findings, with ICERs of -€75,508/QALY in the *HER2^+^* (non-luminal) group and -€83,707/QALY in the Luminal B group.

**Table 7:** Cost-effectiveness analysis.

Parameters	Patients (*n)*	Cost (€)	QALYs	ICER (€/QALY)
*HER2*^+^breast cancers				
Adjuvant trastuzumab-treated	151	43,682	2.194	(reference)
Neoadjuvant trastuzumab-treated	28	50,093	2.112	−78,183 (dominated)
NACT/ACT trastuzumab-treated	13	45,367	2.170	−70,208 (dominated)
*HER2^+^*(non-luminal)				
Adjuvant trastuzumab	60	45,639	2.108	(reference)
Neoadjuvant trastuzumab	12	50,094	2.049	−75,508 (dominated)
Luminal B				
Adjuvant trastuzumab	91	42,391	2.252	(reference)
Neoadjuvant trastuzumab	16	50,092	2.160	−83,707 (dominated)

All costs are reported in Euros (€), valued at 2010–2012 prices. 95% CI: 95% confidence interval; ACERs: Average cost-effectiveness ratios; ACT: Adjuvant chemotherapy; *HER2*: Human epidermal growth factor receptor 2; ICER: Incremental cost-effectiveness ratio; NACT: Neoadjuvant chemotherapy; QALYs: Quality-adjusted life years.

Sensitivity analysis demonstrated that the NACT/ACT trastuzumab regimen remained cost-effective under all tested utility scenarios, with ICERs ranging from –€176,314 to €37,772 per QALY. In contrast, the neoadjuvant-only strategy demonstrated substantial uncertainty, with ICERs ranging from –€670,829 to €143,712 per QALY, reflecting the instability of estimates driven by the very small QALY differences. While many estimates were either dominated or fell below the Irish cost-effectiveness threshold of €45,000 per QALY, the wide variation highlights the strong influence of utility assumptions on cost-effectiveness outcomes.

## Discussion

In this study, we reported the costs and health outcomes associated with the treatment of *HER2^+^* breast cancers with either adjuvant, neoadjuvant, or NACT/ACT trastuzumab regimens and explored variations in cost-effectiveness between groups. Although not designed as a health economic assessment, our cost-effectiveness findings provide important insights. We confirmed our hypothesis, that the use of neoadjuvant trastuzumab did not result in a significant economic disadvantage. Given that the adjuvant and neoadjuvant groups demonstrated comparable QALY and DFS, these results support a wider move to use many established adjuvant therapeutics in the neoadjuvant setting. As neoadjuvant therapy continues to gain prominence as a clinically effective treatment option,^36^ our findings may inform policy development in populations experiencing rising cancer incidence, where neoadjuvant chemotherapy use is being increasingly adopted.^37^^,^^38^

Our analysis supports previous work demonstrating variation in survival and outcomes between the two *HER2*-positive breast cancer subtypes.^25^ To explore this further, we investigated potential differences in treatment costs by subtype (Luminal B *HER2* and *HER2^+^*[nonluminal]), clinicopathological features (treatment type, surgical procedure, age, disease stage, tumour grade), and QALYs. In our study, no significant difference in direct treatment cost was seen between the adjuvant trastuzumab or neoadjuvant trastuzumab groups (€43,682 vs €50,093). Multivariate analysis found that neoadjuvant trastuzumab treatment was not a significant factor in driving treatment costs to increase. It should be noted, however, that these estimates are conservative given the 3-year follow-up timeframe, and extended follow-up is warranted to assess long-term cost dynamics for patients surviving beyond this key observation period. Our exploratory cost-effectiveness analysis indicated that adjuvant trastuzumab is likely the most cost-effective strategy. Nonetheless, a formal health economic assessment conducted following the HIQA guidelines would be required to definitively address the question of the relative cost-effectiveness of trastuzumab in the neoadjuvant setting, as it becomes more widely adopted.

Previous studies demonstrated that the addition of adjuvant trastuzumab to the treatment of *HER2^+^* breast cancers significantly improved survival and reduced recurrence.^26^^,^^39^ Importantly, adjuvant trastuzumab treatment was shown to be associated with higher quality-adjusted life expectancy by 1.54 QALYs.^40^ Numerous studies have assessed the cost-effectiveness of adjuvant trastuzumab treatment in *HER2*-positive breast cancer, with the majority of these studies suggesting it would be cost-effective, at less than the often quoted (American) cost-effectiveness threshold of $50,000/QALY^20^ and the Irish threshold of €45,000/QALY.^41^ In the current study, the potential cost-effectiveness of adjuvant trastuzumab treatment is once again confirmed. However, there are issues in using cost/QALY for the analysis of the cost-effectiveness of trastuzumab treatment. A systematic review of cost-effectiveness found large variation in cost per QALY between studies, ranging from $5020/QALY to $134,610/QALY, and almost 85% of studies used a predictive Markov model to obtain their results.^42^ The large variation in reported cost per QALY may be due to publication bias, where studies only publish positive or negative, but not intermediate, results.^43^ Our exploratory cost-effectiveness analysis found that the neoadjuvant trastuzumab was dominated by adjuvant trastuzumab; however, the differences in mean costs and mean QALYs between the two treatment approaches were minimal. The ICER estimates, particularly for the neoadjuvant group, showed substantial variability, highlighting the need for cautious interpretation and further research. Importantly, the modest economic differences between neoadjuvant trastuzumab and adjuvant trastuzumab represent a key consideration for healthcare systems, where robust economic evidence—such as for the use of neoadjuvant therapy in breast cancer—is increasingly needed to inform policy decisions in the face of an ageing population and a growing cancer burden.^36^^,^^44^

The addition of neoadjuvant trastuzumab treatment to one year of adjuvant trastuzumab treatment has been shown to improve DFS.^29^ Furthermore, the addition of neoadjuvant trastuzumab treatment to chemotherapy regimens for *HER2*-positive breast cancers has been shown to significantly improve pCR rates.^33^^,^^34^ However, pooled analysis of prospective neoadjuvant chemotherapy trials demonstrated that survival correlates closely with treatment response, with patients having a pCR also having an improved DFS.^45^ In our study with 3 years of follow-up data, no significant difference was seen in survival between the adjuvant and neoadjuvant trastuzumab treatment groups on multivariate analysis.

Analysis of the adjuvant trastuzumab-treated group found no significant differences in cost, either overall or when stratified by *HER2* subtype. In contrast, within the neoadjuvant trastuzumab-treated group, a non-significant reduction in cost was observed in the *HER2^+^* (non-luminal) subtype. Previous work suggests this could be attributed to the higher rates of pCR seen in *HER2^+^*(non-luminal) subtypes,^45^^,^^46^ which led to fewer surgical interventions and lower recurrence rates. However, additional factors may influence the decision to include neoadjuvant trastuzumab in treatment regimens. The EBCTCG meta-analysis highlighted that neoadjuvant therapy is not yet universally adopted and may be associated with a moderate increase in local recurrence when combined with breast conserving surgery.^47^, ^48^, ^49^

Several factors limited the ability to conduct a more comprehensive cost analysis. These include the retrospective observational nature of the study, incomplete or difficult-to-cost data—particularly where double counting was a risk— and significant variability in factors like the length of hospital stay or the need for additional in-hospital treatments (e.g., ICU admission). Additionally, a substantial proportion of the neoadjuvant-treated patients had late-stage or node-positive disease, which increased the likelihood of requiring mastectomy and axillary node clearance. These clinical factors are associated with a higher risk of disease recurrence and likely contributed to the higher costs observed in the neoadjuvant trastuzumab-treated group, with later disease stage found to be a significant driver of increased cost in the multivariate analysis. Moreover, as the DFS rate was calculated at three years follow-up, longer-term follow-up may offer a more accurate evaluation of the cost-effectiveness of neoadjuvant trastuzumab. Another limitation is that the costing estimates were based on pricing at the time of treatment, without any adjustment for inflation; thus, these figures should not be used for current cost-effectiveness decisions. Future studies should incorporate inflation-adjusted cost estimates. Finally, while the relatively small size of the neoadjuvant group limits the statistical power to detect modest cost differences, the analysis nonetheless offers valuable insights, particularly in the comparisons by therapy type and tumour subtype.

In conclusion, when comparing costs and health outcomes, no significant differences were observed between the neoadjuvant and adjuvant trastuzumab groups, nor across *HER2*-positive breast cancer subtypes. Although this study does not definitively establish the relative cost-effectiveness of these treatment approaches, it provides valuable evidence to inform future studies and policy development. The findings support the increasing adoption of neoadjuvant chemotherapy, indicating no substantial economic disadvantage associated with its use. Moreover, this study indicates that investing in neoadjuvant trastuzumab may reduce treatment costs and improve patient outcomes. This has important implications in healthcare planning, particularly in the context of an ageing cancer population and the increasing need for efficient, evidence-based resource allocation.

## Authors contribution

Conceptualization: James A.L. Brown, Michael J. Kerin; Methodology: Amirhossein Jalali, Shirin Moghaddam, Andrew McGuire, Doireann P. Joyce, James A.L. Brown, Ciaran O'Neill; Formal analysis: Amirhossein Jalali, Shirin Moghaddam, James A.L. Brown, Ciaran O'Neill; Writing - Original Draft: Amirhossein Jalali, Shirin Moghaddam, James A.L. Brown; Writing - Review & Editing: Amirhossein Jalali, Shirin Moghaddam, James A.L. Brown, Ciaran O'Neill, Paddy Gillespie, Bridget Carr, Diarmuid O'Leary, Emer Bourke; Supervision: James A.L. Brown; Funding: James A.L. Brown, Michael J. Kerin. All the authors critically revised and approved the final version of the manuscript.

## Ethics statement

This study was conducted in accordance with the granted ethical approval from the University Hospital Galway and National University of Ireland Galway (C.A.151 and C.A.1012) and adheres to the *Declaration of Helsinki*. All patients’ clinic-pathological data were obtained from a prospectively maintained anonymized database extracted from electronic and hard copies of patient records and covered the active period of care with a minimum of 12 months postdiagnosis. Informed consent was waived, given the retrospective nature of the study.

## Data availability statement

Anonymized patient data and treatment costings utilised in this study are available upon request (colette.collins@hse.ie) for researchers who meet the criteria for access to confidential data.

## Declaration of Generative AI and AI-assisted technologies in the writing process

The authors declare that no generative artificial intelligence (AI) or AI-assisted technologies were used in the writing, or any other process, for the preparation of this manuscript.

## Funding

This work was supported by funding from the National Breast Cancer Research Institute (Ireland).

## Conflict of interest

The authors declare that they have no known competing financial interests or personal relationships that could have appeared to influence the work reported in this paper.
